# Hazards of Cold Water—Paul Esser Memorial Lecture 1973

**Published:** 1973-07

**Authors:** W. R. Keatinge

**Affiliations:** Professor of Physiology at the London Hospital


### The Paul Esser Memorial Lecture 1973

This is an annual lecture given in the University of Bristol on some subject connected with one of the water sports, caving or mountaineering. It is in memory of Paul Esser, a medical student who lost his life while cave diving in Porth yr Ogof in February of 1971, and who was particularly interested in those sports. The lecturer is chosen by a panel of representatives from the student societies in the University that are interested in these sports, together with the Cave Diving Group and the University Department of Physical Education. This year's Lecture was given on 14th February 1973 on the subject of

### HAZARDS OF COLD WATER

By

Professor W. R. Keatinge, Professor of Physiology at the London Hospital

Professor Keatinge was studying the cardiac and respiratory reflexes to cold water on the skin in America during 1963/4 and so became well qualified to study the effects of the *Lakonia* disaster of December 1963. This ship caught fire in the Mediterranean and was abandoned by its crew and passengers, a large number of whom died in the water. Professor Keatinge was able to show that the principal cause of death was not drowning so much as cold exposure.

He continued his study of the effect of cold on survival at Oxford and at the London Hospital, and in 1969 drew attention to some of the reasons why people were unable to swim in cold water.

Professor Keatinge pointed out that some of the most exciting sports are the dangerous ones, but the risk becomes quite small if the hazards are understood. Of all these, water sports take the greatest toll of human life: about 1,000 deaths in a year around Great Britain, compared with about a dozen on the mountains. Death from shipwreck results more often from cold than from drowning. Old-fashioned equipment was designed to provide flotation rather than to protect from cold, but ideally both should be provided. Since the last war much research has been devoted to studying body-cooling of volunteers, measuring core temperature by electric thermometers. These give most reliable measurements when swallowed to lie just behind the heart.

### Body Cooling

Thin men cool faster than fat men, because the layer of fat insulates the body core. The cold causes the blood vessels in the fat to shut down. Channel swimmers are usually fat. The usual summer sea temperature here is about 15°C. At this temperature fat men have a distinct advantage over thin. For thin men the critical water temperature at which heat balance is possible is about 20°C, for fat it is 10°C. Below these temperatures the rate of body-cooling is uncontrollable, even by shivering. The rate of body cooling can, however, be reduced, firstly by keeping still, because exercise in water (unlike air) always accelerates body-cooling if the water is cold enough to threaten life, and, secondly, by keeping on as much clothing as possible, as this will slow down the rate of cooling.

The same principles apply to children, who often seem to be able to tolerate cold water better than adults. This is an illusion. They cool more rapidly, both because they are usually thinner and because they have a larger surface area of skin in relation to body weight. Girls generally cool more slowly than boys because they are fatter. In one experiment one boy cooled as much as 3.2°C in 33 minutes. All the children who looked really cold were found to have core temperatures of less than 35°C (normal is 37°C), which is a fairly serious degree of hypothermia.

### Cold Vasodilatation

At temperatures near freezing point the protective shut-down of blood vessels in skin becomes reversed due to paralysis of their muscular walls. The resulting vasodilatation accelerates cooling, particularly of the hands from which heat pours out of the body. The practical answer to this is a "wet suit" with gloves. Whales and seals do not get cold vasodilatation at low temperatures and so their blubber will always protect them from heat loss.

### Sudden Sinking

Professor Keatinge quoted the case of a young athlete out sailing in the winter on a reservoir, when his boat overturned. He only had 50 yards to swim to the shore, but after he had got half way he shouted that he couldn't go on and sank. Cramp could be ruled out as he was in good training. Study of skin reflexes to cold shows that respiration is accelerated, and air is not expelled from the chest as fully as usual. The heart accelerates, the cardiac output doubles and the blood pressure goes up. Possibly the overbreathing in choppy water might cause water to be inhaled; but a more important finding was that irregularities of heart beat occurred (ectopic ventricular beats). These occurred in 10 to 15% of subjects on being immersed in cold water. After a few minutes these irregularities diminish because the nerve endings in the skin adapt to the cold. None of these things accounted for the sudden sinking of the lad in the reservoir, so it became necessary to design an experiment under controlled conditions, which would simulate the circumstances.

This was done by a good swimmer dressing up, getting into water at 4.7°C and starting to swim; within 90 seconds he sank and had to be pulled out. A film was shown of another swimmer repeating the experiment. First he was overbreathing, due to the cold water on the skin. When he began swimming he was holding his head high out of the water, which is tiring. He quickly began to get exhausted and to make small mistakes. After 7½ minutes he had to stop and be pulled out. On the bank he was utterly exhausted but within 1½ minutes was talking cheerfully. "I don't know why I couldn't", he said, "I just got exhausted; I couldn't go on".

The explanation why cold water is more exhausting than hot is very simple and has nothing to do with hypothermia. This man had no drop in core temperature. It is that cold water is stickier (more viscous) than warm; it is like trying to swim in treacle.

**Practical Advice**

Prof. Keatinge gave the following counsel. Always wear a life jacket when sailing. If you have to abandon ship on the open sea make sure that you are fully clothed (or wear a wet suit), don't exercise yourself but keep afloat until rescued. The natural thing to do is to swim about. This is a case where the natural thing is the wrong thing to do. If you are caught in cold water without a life jacket do not swim for shore; cling to the boat until rescued if rescue is on the way.

When a subject appears to have died from hypothermia, do not despair. Plunge him if possible into a bath of water as hot as the hand can stand. This is a life-saving measure if done early. The hot bath should not be continued after a satisfactory heart beat and respiration are restored.

Alcohol taken before immersion in cold water does not noticeably accelerate heat loss and makes the ordeal more tolerable. On the other hand, if taken after two hours of exhausting exercise, it can cause a dramatic fall in blood sugar, which eliminates the ability of the body to control temperature, and so may be lethal. So if you take a hip flask up a mountain, take some sweets as well!

